# Upper extremity thromboses in medical-surgical critically ill patients

**DOI:** 10.1186/cc9442

**Published:** 2011-03-11

**Authors:** N Zytaruk, F Lamontagne, L McIntyre, P Dodek, N Vlahakis, B Lewis, D Schiff, A Moody, M Ostermann, S Padayachee, D Heels-Ansdell, S Vallance, A Davies, JD Cooper, DJ Cook

**Affiliations:** 1 McMaster University, Hamilton, Canada; 2 Sherbrooke Hospital, Quebec, Canada; 3 Ottawa Health Research Institute, Ottawa, Canada; 4 St Paul's Hospital, Vancouver, Canada; 5 Mayo Clinic, Rochester, MN, USA; 6 Sunnybrook Health Science Center, Toronto, Canada; 7 Guy's & St Thomas' Hospital, London, UK; 8 Alfred Hospital, Melbourne, Australia

## Introduction

Venous thrombosis of the upper extremity is a recognized complication of critical illness. The objective of this study was to describe the incidence and characteristics of upper-extremity thromboses in patients who were enrolled in an international trial that compared UFH versus LMWH as prophylaxis for VTE (NCT00182143).

## Methods

We recorded the location, extent and prior catheterization of all patients who had upper-extremity venous thromboses confirmed by compression ultrasonography or computed tomography. No patients were routinely screened for upper-extremity thromboses. We excluded prevalent thromboses found within 72 hours of ICU admission. If a patient had both deep and superficial thromboses, we categorized as deep; if a patient had both proximal and distal thromboses, we categorized as proximal. We defined catheter-related thromboses as partial or complete noncompressibility of the same or a contiguous segment in which a catheter had been inserted within the previous 72 hours. Events were adjudicated in duplicate by physicians blinded to study drug and each others' assessments.

## Results

Among 3,659 patients, 72 (2.0%) developed upper extremity thrombosis involving 129 unique venous segments. Of 72 patients, 35 (48.6%) patients had thromboses in more than one segment. Most thromboses (86, 66.7%) were on the right side. Most of these were deep (56, 77.8%), but a few were superficial (16, 22.2%). Most had proximal thromboses (65, 90.3%), but a few had distal (7, 9.7%). The three commonest sites of thrombosis were the internal jugular (29.5%), subclavian (18.6%) and cephalic (17.8%) veins. Less commonly affected were the brachial (12.4%), axillary (8.5%), basilic (8.5%), innominate (3.9%) and external jugular (0.8%) veins. Overall, 69 (53.5%) thromboses were catheter-related.

## Conclusions

In medical-surgical patients who are receiving heparin prophylaxis, upper extremity DVT was uncommon, occurring in 2% of patients. These thromboses may be clinically important, because the majority is proximal and three-quarters are deep. Revisiting the need for central vascular access daily is underscored by the finding that half were catheter-related.

